# 1-Methyl-3-(2-methyl­phen­yl)-3,3a,4,9b-tetra­hydro-1*H*-chromeno[4,3-*c*][1,2]oxazole-3a-carbonitrile

**DOI:** 10.1107/S1600536811009378

**Published:** 2011-03-19

**Authors:** K. Swaminathan, K. Sethusankar, G. Murugan, M. Bakthadoss

**Affiliations:** a Department of Physics, RKM Vivekananda College (Autonomous), Chennai 600 004, India; b Department of Organic Chemistry, University of Madras, Maraimalai Campus, Chennai 600 025, India

## Abstract

In the title compound, C_19_H_18_N_2_O_2_, the five-membered isoxazole ring adopts an envelope conformation and the deviation of the N atom from the mean plane of the isoxazole ring is −0.3256 (11) Å. The pyran ring adopts a half-chair conformation. The isoxazole ring forms dihedral angles of 44.07 (7) and 84.23 (7)° with the pyran and methyl­benzene rings, respectively. The mol­ecular structure is stabilized by weak C—H⋯π inter­actions.

## Related literature

For the synthesis of the title compound, see: Bakthadoss & Murugan (2010 ▶). For the biological and pharmacological activities of isoxazole derivatives, see: Hu *et al.* (2004 ▶); Lin *et al.* (1996 ▶); Rozman *et al.* (2002 ▶). For a related structure, see: Swaminathan *et al.* (2011 ▶). For puckering amplitudes, see: Cremer & Pople (1975 ▶).
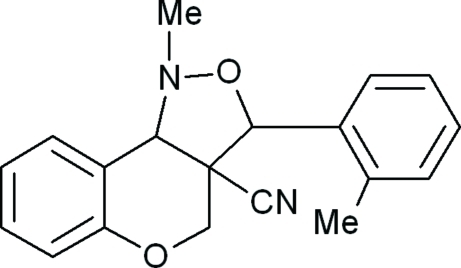


## Experimental

### 

#### Crystal data

C_19_H_18_N_2_O_2_
*M* *_r_* = 306.35
Monoclinic, 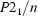

*a* = 11.0120 (4) Å
*b* = 13.0368 (4) Å
*c* = 11.1977 (3) Å
β = 97.836 (2)°
*V* = 1592.54 (9) Å^3^
*Z* = 4
Mo *K*α radiation
μ = 0.08 mm^−1^
*T* = 293 K
0.30 × 0.25 × 0.20 mm

#### Data collection

Bruker Kappa APEXII CCD diffractometer
23069 measured reflections
5936 independent reflections
3084 reflections with *I* > 2σ(*I*)
*R*_int_ = 0.035

#### Refinement

*R*[*F* ^2^ > 2σ(*F* ^2^)] = 0.051
*wR*(*F* ^2^) = 0.148
*S* = 1.01
5936 reflections
210 parameters
H-atom parameters constrained
Δρ_max_ = 0.18 e Å^−3^
Δρ_min_ = −0.21 e Å^−3^

### 

Data collection: *APEX2* (Bruker, 2004 ▶); cell refinement: *SAINT* (Bruker, 2004 ▶); data reduction: *SAINT*; program(s) used to solve structure: *SHELXS97* (Sheldrick, 2008 ▶); program(s) used to refine structure: *SHELXL97* (Sheldrick, 2008 ▶); molecular graphics: *ORTEP-3* (Farrugia, 1997 ▶); software used to prepare material for publication: *SHELXL97* and *PLATON* (Spek, 2009 ▶).

## Supplementary Material

Crystal structure: contains datablocks global, I. DOI: 10.1107/S1600536811009378/pv2397sup1.cif

Structure factors: contains datablocks I. DOI: 10.1107/S1600536811009378/pv2397Isup2.hkl

Additional supplementary materials:  crystallographic information; 3D view; checkCIF report

## Figures and Tables

**Table 1:** Hydrogen-bond geometry (Å, °) *Cg*1 is the centroid of the C11–C16 phenyl ring.

*D*—H⋯*A*	*D*—H	H⋯*A*	*D*⋯*A*	*D*—H⋯*A*
C18—H18*B*⋯*Cg*1^i^	0.96	2.99	3.6831 (16)	130

Symmetry code: (i) 
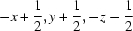
.

**Fig. 1.**
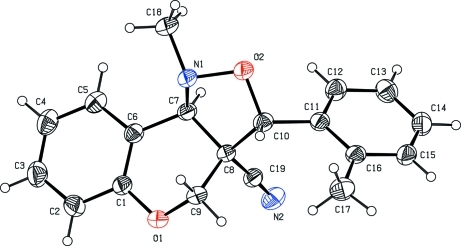
The molecular structure of the title compound with the atom numbering scheme. Displacement ellipsoids are drawn at 30% probability level. H atoms are drawn as small spheres of arbitary radius.

## supplementary crystallographic information

### Comment

The title compound is an angularly substituted fused tricyclic chromenoisoxazolidine framework, synthesized using Baylis-Hillman derivatives through *in situ* formation of nitrones followed by an intramolecular (3 + 2) dipolar cycloaddition reaction sequence (Bakthadoss & Murugan, 2010). It is well known that benzopyran and isoxazolidine derivatives possess interesting biological and pharmacological activities (Lin *et al.*, 1996; Hu *et al.*, 2004). Leflunomide is an isoxazole drug used for the treatment of rheumatoid arthritis (Rozman *et al.*, 2002).

The title compound (Fig. 1) comprises a chromenoisoxazole ring system attached to a methylbenzene ring and a carbonitrile group. The isoxazole ring (N1/O2/C7/C8/C10) adopts an N1 envelope conformation with N1 -0.3256 (11) Å out of the plane formed by the rest of the ring atoms. The isoxazole ring is inclined at 84.23 (7)° with respect to the mean-plane formed by methylbenzene ring (C11—C16). The pyran ring (O1/C1/C6-C9) adopts a half-chair conformation with puckering amplitudes (Cremer & Pople, 1975): *Q* = 0.4852 (13) Å, θ = 127.2 (2)° and φ = 107.9 (2)°. The title compound exhibits structural similarities with a reported structure (Swaminathan *et al.*, 2011). The molecular structure of the title compound is stabilized by rather weak C18—H18B···π interactions involving the centroid (Cg1) of phenyl ring C11–C16 (Table 1).

### Experimental

A mixture of (*E*)-2-((2-formylphenoxy) methyl) -3-*o*-tolylacrylonitrile (1.0 mmol), *N*-methylhydroxylamine hydrochloride (1.1 mmol), pyridine (0.24 ml, 3 mmol) and ethanol (5 ml) were placed in a round bottom flask and refluxed for 6 h. After completion of the reaction as indicated by TLC, the reaction mixture was concentrated under reduced pressure. The crude product was diluted with water (10 ml) and dil HCl (5 ml) and extracted with ethylacetate (20 ml). The organic layer was washed with brine solution (10 ml) and concetrated. The crude product was purified by column chromatography to provide the pure title compound, as a colourless solid. Crystals of the title compound were grown from its solution in methanol by slow evaporation at room temperature.

### Refinement

Hydrogen atoms were placed in calculated positions with C—H = 0.93, 0.96, 0.97 and 0.98 Å for aryl, methyl, methylene and methyne type H-atoms, respectively, and refined in riding model with fixed isotropic displacement parameters: *U*_iso_(H) = 1.5 *U*_eq_(methyl-C) and *U*_iso_(H) = 1.2 *U*_eq_(the rest of the C atoms).

### Crystal data

**Table:**

C_19_H_18_N_2_O_2_	*F*(000) = 648
*M**_r_* = 306.35	*D*_x_ = 1.278 Mg m^−^^3^
Monoclinic, *P*2_1_/*n*	Mo *K*α radiation, λ = 0.71073 Å
Hall symbol: -p 2yn	Cell parameters from 5936 reflections
*a* = 11.0120 (4) Å	θ = 1.0–25°
*b* = 13.0368 (4) Å	µ = 0.08 mm^−^^1^
*c* = 11.1977 (3) Å	*T* = 293 K
β = 97.836 (2)°	Block, colourless
*V* = 1592.54 (9) Å^3^	0.30 × 0.25 × 0.20 mm
*Z* = 4	

### Data collection

**Table:**

Bruker Kappa APEXII CCD diffractometer	3084 reflections with *I* > 2σ(*I*)
Radiation source: fine-focus sealed tube	*R*_int_ = 0.035
graphite	θ_max_ = 33.1°, θ_min_ = 2.4°
ω scans	*h* = −15→16
23069 measured reflections	*k* = −18→19
5936 independent reflections	*l* = −14→17

### Refinement

**Table:**

Refinement on *F*^2^	Primary atom site location: structure-invariant direct methods
Least-squares matrix: full	Secondary atom site location: difference Fourier map
*R*[*F*^2^ > 2σ(*F*^2^)] = 0.051	Hydrogen site location: inferred from neighbouring sites
*wR*(*F*^2^) = 0.148	H-atom parameters constrained
*S* = 1.01	*w* = 1/[σ^2^(*F*_o_^2^) + (0.0684*P*)^2^ + 0.0556*P*] where *P* = (*F*_o_^2^ + 2*F*_c_^2^)/3
5936 reflections	(Δ/σ)_max_ < 0.001
210 parameters	Δρ_max_ = 0.18 e Å^−^^3^
0 restraints	Δρ_min_ = −0.21 e Å^−^^3^

### Special details

**Table:**

Geometry. All e.s.d.'s (except the e.s.d. in the dihedral angle between two l.s. planes) are estimated using the full covariance matrix. The cell e.s.d.'s are taken into account individually in the estimation of e.s.d.'s in distances, angles and torsion angles; correlations between e.s.d.'s in cell parameters are only used when they are defined by crystal symmetry. An approximate (isotropic) treatment of cell e.s.d.'s is used for estimating e.s.d.'s involving l.s. planes.
Refinement. Refinement of *F*^2^ against ALL reflections. The weighted *R*-factor *wR* and goodness of fit *S* are based on *F*^2^, conventional *R*-factors *R* are based on *F*, with *F* set to zero for negative *F*^2^. The threshold expression of *F*^2^ > σ(*F*^2^) is used only for calculating *R*-factors(gt) *etc*. and is not relevant to the choice of reflections for refinement. *R*-factors based on *F*^2^ are statistically about twice as large as those based on *F*, and *R*- factors based on ALL data will be even larger.

### Fractional atomic coordinates and isotropic or equivalent isotropic displacement parameters (Å^2^)

**Table:**

	*x*	*y*	*z*	*U*_iso_*/*U*_eq_	
C1	0.38107 (11)	0.12807 (10)	0.10789 (11)	0.0446 (3)	
C2	0.43271 (13)	0.21739 (11)	0.15852 (13)	0.0582 (4)	
H2	0.4706	0.2178	0.2380	0.070*	
C3	0.42782 (15)	0.30530 (12)	0.09094 (15)	0.0654 (4)	
H3	0.4635	0.3652	0.1245	0.078*	
C4	0.37052 (14)	0.30563 (12)	−0.02619 (15)	0.0640 (4)	
H4	0.3685	0.3653	−0.0720	0.077*	
C5	0.31626 (13)	0.21749 (10)	−0.07520 (13)	0.0536 (3)	
H5	0.2754	0.2187	−0.1535	0.064*	
C6	0.32143 (11)	0.12627 (9)	−0.00950 (10)	0.0425 (3)	
C7	0.25834 (11)	0.03122 (9)	−0.06218 (10)	0.0407 (3)	
H7	0.1745	0.0473	−0.0987	0.049*	
C8	0.25759 (10)	−0.05625 (9)	0.02964 (10)	0.0396 (3)	
C9	0.37624 (12)	−0.05094 (10)	0.11822 (11)	0.0458 (3)	
H9A	0.3776	−0.1068	0.1756	0.055*	
H9B	0.4458	−0.0593	0.0744	0.055*	
C10	0.25780 (11)	−0.15460 (10)	−0.05147 (10)	0.0448 (3)	
H10	0.3344	−0.1923	−0.0288	0.054*	
C11	0.15148 (11)	−0.22621 (9)	−0.04883 (10)	0.0430 (3)	
C12	0.03836 (12)	−0.19982 (11)	−0.11130 (12)	0.0528 (3)	
H12	0.0313	−0.1411	−0.1589	0.063*	
C13	−0.06361 (13)	−0.25916 (12)	−0.10388 (13)	0.0577 (4)	
H13	−0.1390	−0.2407	−0.1462	0.069*	
C14	−0.05336 (13)	−0.34544 (11)	−0.03398 (13)	0.0571 (4)	
H14	−0.1221	−0.3854	−0.0278	0.069*	
C15	0.05804 (14)	−0.37299 (10)	0.02683 (12)	0.0544 (3)	
H15	0.0638	−0.4322	0.0735	0.065*	
C16	0.16284 (12)	−0.31509 (10)	0.02085 (10)	0.0458 (3)	
C17	0.28229 (15)	−0.35091 (13)	0.08907 (14)	0.0714 (4)	
H17A	0.3372	−0.3698	0.0331	0.107*	
H17B	0.2679	−0.4092	0.1376	0.107*	
H17C	0.3181	−0.2966	0.1400	0.107*	
C18	0.31258 (15)	0.02789 (12)	−0.26883 (11)	0.0624 (4)	
H18A	0.3441	−0.0171	−0.3251	0.094*	
H18B	0.3574	0.0912	−0.2641	0.094*	
H18C	0.2275	0.0413	−0.2953	0.094*	
C19	0.15272 (11)	−0.05003 (9)	0.09694 (11)	0.0439 (3)	
N1	0.32585 (9)	−0.02011 (8)	−0.15097 (8)	0.0464 (3)	
N2	0.07493 (11)	−0.04521 (10)	0.15340 (10)	0.0598 (3)	
O1	0.38708 (8)	0.04332 (7)	0.18095 (7)	0.0501 (2)	
O2	0.25538 (9)	−0.11520 (7)	−0.17000 (7)	0.0540 (3)	

### Atomic displacement parameters (Å^2^)

**Table:**

	*U*^11^	*U*^22^	*U*^33^	*U*^12^	*U*^13^	*U*^23^
C1	0.0477 (6)	0.0468 (7)	0.0411 (6)	−0.0039 (5)	0.0120 (5)	−0.0003 (5)
C2	0.0646 (8)	0.0602 (9)	0.0508 (7)	−0.0107 (7)	0.0116 (6)	−0.0100 (7)
C3	0.0768 (10)	0.0492 (9)	0.0737 (10)	−0.0142 (7)	0.0224 (8)	−0.0106 (8)
C4	0.0764 (10)	0.0434 (8)	0.0756 (10)	−0.0011 (7)	0.0229 (8)	0.0065 (7)
C5	0.0592 (8)	0.0497 (8)	0.0530 (7)	0.0026 (6)	0.0116 (6)	0.0084 (6)
C6	0.0446 (6)	0.0427 (7)	0.0416 (6)	0.0008 (5)	0.0116 (5)	0.0013 (5)
C7	0.0426 (6)	0.0460 (7)	0.0341 (5)	−0.0010 (5)	0.0071 (4)	0.0045 (5)
C8	0.0436 (6)	0.0430 (7)	0.0332 (5)	−0.0024 (5)	0.0089 (4)	0.0016 (5)
C9	0.0518 (7)	0.0487 (7)	0.0370 (6)	−0.0009 (6)	0.0060 (5)	0.0052 (5)
C10	0.0519 (7)	0.0451 (7)	0.0392 (6)	0.0005 (5)	0.0128 (5)	−0.0001 (5)
C11	0.0529 (7)	0.0397 (7)	0.0379 (5)	−0.0008 (5)	0.0121 (5)	−0.0041 (5)
C12	0.0604 (8)	0.0440 (7)	0.0544 (7)	0.0017 (6)	0.0087 (6)	0.0031 (6)
C13	0.0516 (7)	0.0597 (9)	0.0617 (8)	−0.0006 (6)	0.0072 (6)	−0.0070 (7)
C14	0.0609 (8)	0.0535 (8)	0.0600 (8)	−0.0130 (7)	0.0192 (7)	−0.0093 (7)
C15	0.0768 (9)	0.0427 (7)	0.0461 (7)	−0.0094 (6)	0.0168 (6)	−0.0025 (6)
C16	0.0623 (8)	0.0398 (7)	0.0360 (6)	−0.0005 (6)	0.0098 (5)	−0.0036 (5)
C17	0.0772 (10)	0.0690 (11)	0.0641 (9)	−0.0002 (8)	−0.0049 (8)	0.0154 (8)
C18	0.0810 (10)	0.0714 (10)	0.0372 (6)	−0.0130 (8)	0.0161 (6)	0.0050 (6)
C19	0.0513 (7)	0.0423 (7)	0.0394 (6)	−0.0030 (5)	0.0102 (5)	0.0016 (5)
N1	0.0557 (6)	0.0498 (6)	0.0361 (5)	−0.0091 (5)	0.0146 (4)	0.0002 (4)
N2	0.0639 (7)	0.0652 (8)	0.0551 (7)	0.0023 (6)	0.0249 (6)	0.0060 (6)
O1	0.0622 (5)	0.0532 (6)	0.0340 (4)	−0.0076 (4)	0.0040 (4)	0.0004 (4)
O2	0.0737 (6)	0.0535 (6)	0.0374 (4)	−0.0161 (4)	0.0166 (4)	−0.0045 (4)

### Geometric parameters (Å, °)

**Table:**

C1—O1	1.3710 (14)	C10—C11	1.5008 (17)
C1—C2	1.3830 (18)	C10—H10	0.9800
C1—C6	1.3872 (17)	C11—C12	1.3864 (18)
C2—C3	1.370 (2)	C11—C16	1.3928 (17)
C2—H2	0.9300	C12—C13	1.3754 (19)
C3—C4	1.376 (2)	C12—H12	0.9300
C3—H3	0.9300	C13—C14	1.366 (2)
C4—C5	1.374 (2)	C13—H13	0.9300
C4—H4	0.9300	C14—C15	1.367 (2)
C5—C6	1.3954 (17)	C14—H14	0.9300
C5—H5	0.9300	C15—C16	1.3882 (18)
C6—C7	1.5015 (17)	C15—H15	0.9300
C7—N1	1.4800 (15)	C16—C17	1.5027 (19)
C7—C8	1.5363 (16)	C17—H17A	0.9600
C7—H7	0.9800	C17—H17B	0.9600
C8—C19	1.4647 (16)	C17—H17C	0.9600
C8—C9	1.5305 (16)	C18—N1	1.4498 (16)
C8—C10	1.5715 (17)	C18—H18A	0.9600
C9—O1	1.4124 (15)	C18—H18B	0.9600
C9—H9A	0.9700	C18—H18C	0.9600
C9—H9B	0.9700	C19—N2	1.1336 (16)
C10—O2	1.4201 (14)	N1—O2	1.4626 (13)
			
O1—C1—C2	116.76 (11)	O2—C10—H10	109.2
O1—C1—C6	122.00 (11)	C11—C10—H10	109.2
C2—C1—C6	121.18 (12)	C8—C10—H10	109.2
C3—C2—C1	119.65 (14)	C12—C11—C16	119.67 (11)
C3—C2—H2	120.2	C12—C11—C10	119.03 (11)
C1—C2—H2	120.2	C16—C11—C10	121.18 (11)
C2—C3—C4	120.50 (14)	C13—C12—C11	120.98 (13)
C2—C3—H3	119.8	C13—C12—H12	119.5
C4—C3—H3	119.8	C11—C12—H12	119.5
C5—C4—C3	119.73 (14)	C14—C13—C12	119.56 (13)
C5—C4—H4	120.1	C14—C13—H13	120.2
C3—C4—H4	120.1	C12—C13—H13	120.2
C4—C5—C6	121.18 (13)	C13—C14—C15	120.00 (13)
C4—C5—H5	119.4	C13—C14—H14	120.0
C6—C5—H5	119.4	C15—C14—H14	120.0
C1—C6—C5	117.72 (12)	C14—C15—C16	121.90 (13)
C1—C6—C7	121.34 (11)	C14—C15—H15	119.1
C5—C6—C7	120.85 (11)	C16—C15—H15	119.1
N1—C7—C6	112.90 (9)	C15—C16—C11	117.88 (12)
N1—C7—C8	99.38 (9)	C15—C16—C17	118.83 (12)
C6—C7—C8	113.21 (9)	C11—C16—C17	123.28 (12)
N1—C7—H7	110.3	C16—C17—H17A	109.5
C6—C7—H7	110.3	C16—C17—H17B	109.5
C8—C7—H7	110.3	H17A—C17—H17B	109.5
C19—C8—C9	109.11 (9)	C16—C17—H17C	109.5
C19—C8—C7	112.33 (9)	H17A—C17—H17C	109.5
C9—C8—C7	108.55 (9)	H17B—C17—H17C	109.5
C19—C8—C10	113.95 (9)	N1—C18—H18A	109.5
C9—C8—C10	110.06 (10)	N1—C18—H18B	109.5
C7—C8—C10	102.61 (9)	H18A—C18—H18B	109.5
O1—C9—C8	111.62 (10)	N1—C18—H18C	109.5
O1—C9—H9A	109.3	H18A—C18—H18C	109.5
C8—C9—H9A	109.3	H18B—C18—H18C	109.5
O1—C9—H9B	109.3	N2—C19—C8	177.11 (13)
C8—C9—H9B	109.3	C18—N1—O2	104.33 (9)
H9A—C9—H9B	108.0	C18—N1—C7	114.76 (11)
O2—C10—C11	109.16 (10)	O2—N1—C7	100.14 (8)
O2—C10—C8	104.11 (9)	C1—O1—C9	114.23 (9)
C11—C10—C8	115.69 (9)	C10—O2—N1	103.20 (8)
			
O1—C1—C2—C3	−178.84 (12)	C9—C8—C10—C11	123.40 (11)
C6—C1—C2—C3	−1.6 (2)	C7—C8—C10—C11	−121.21 (11)
C1—C2—C3—C4	0.9 (2)	O2—C10—C11—C12	−41.16 (15)
C2—C3—C4—C5	0.9 (2)	C8—C10—C11—C12	75.80 (14)
C3—C4—C5—C6	−2.0 (2)	O2—C10—C11—C16	142.76 (11)
O1—C1—C6—C5	177.60 (11)	C8—C10—C11—C16	−100.28 (13)
C2—C1—C6—C5	0.56 (18)	C16—C11—C12—C13	1.17 (19)
O1—C1—C6—C7	0.98 (18)	C10—C11—C12—C13	−174.97 (12)
C2—C1—C6—C7	−176.06 (11)	C11—C12—C13—C14	0.0 (2)
C4—C5—C6—C1	1.29 (19)	C12—C13—C14—C15	−0.8 (2)
C4—C5—C6—C7	177.93 (12)	C13—C14—C15—C16	0.5 (2)
C1—C6—C7—N1	−106.66 (12)	C14—C15—C16—C11	0.60 (19)
C5—C6—C7—N1	76.83 (14)	C14—C15—C16—C17	−179.31 (13)
C1—C6—C7—C8	5.29 (16)	C12—C11—C16—C15	−1.42 (17)
C5—C6—C7—C8	−171.23 (10)	C10—C11—C16—C15	174.63 (11)
N1—C7—C8—C19	−152.86 (9)	C12—C11—C16—C17	178.48 (13)
C6—C7—C8—C19	87.13 (12)	C10—C11—C16—C17	−5.46 (18)
N1—C7—C8—C9	86.40 (10)	C6—C7—N1—C18	−77.73 (13)
C6—C7—C8—C9	−33.60 (13)	C8—C7—N1—C18	162.04 (10)
N1—C7—C8—C10	−30.08 (10)	C6—C7—N1—O2	171.21 (9)
C6—C7—C8—C10	−150.08 (10)	C8—C7—N1—O2	50.98 (10)
C19—C8—C9—O1	−62.50 (12)	C2—C1—O1—C9	−157.77 (11)
C7—C8—C9—O1	60.20 (12)	C6—C1—O1—C9	25.06 (16)
C10—C8—C9—O1	171.77 (9)	C8—C9—O1—C1	−56.41 (13)
C19—C8—C10—O2	120.23 (10)	C11—C10—O2—N1	157.42 (9)
C9—C8—C10—O2	−116.84 (10)	C8—C10—O2—N1	33.34 (11)
C7—C8—C10—O2	−1.45 (11)	C18—N1—O2—C10	−173.44 (10)
C19—C8—C10—C11	0.47 (15)	C7—N1—O2—C10	−54.44 (11)

### Hydrogen-bond geometry (Å, °)

**Table:**

Cg1 is the centroid of the phenyl ring C11–C16.

**Table:**

*D*—H···*A*	*D*—H	H···*A*	*D*···*A*	*D*—H···*A*
C18—H18B···Cg1^i^	0.96	2.99	3.6831 (16)	130

Symmetry codes: (i) −*x*+1/2, *y*+1/2, −*z*−1/2.
